# Gephyrin-Independent GABA_A_R Mobility and Clustering during Plasticity

**DOI:** 10.1371/journal.pone.0036148

**Published:** 2012-04-26

**Authors:** Fumihiro Niwa, Hiroko Bannai, Misa Arizono, Kazumi Fukatsu, Antoine Triller, Katsuhiko Mikoshiba

**Affiliations:** 1 Laboratory for Developmental Neurobiology, Brain Science Institute (BSI), RIKEN, Saitama, Japan; 2 Laboratory of Functional Genomics, Department of Medical Genome Science, Graduate School of Frontier Science, The Institute of Medical Science, The University of Tokyo, Minato-ku, Tokyo, Japan; 3 Division of Neuronal Network, The Institute of Medical Science, The University of Tokyo, Tokyo, Japan; 4 Institut de Biologie de l'École Normale Supérieure (IBENS), Institut National de la Santé et de la Recherche Médicale U1024, Centre National de la Recherche Scientifique UMR8197, Paris, France; Institute for Interdisciplinary Neuroscience, France

## Abstract

The activity-dependent modulation of GABA-A receptor (GABA_A_R) clustering at synapses controls inhibitory synaptic transmission. Several lines of evidence suggest that gephyrin, an inhibitory synaptic scaffold protein, is a critical factor in the regulation of GABA_A_R clustering during inhibitory synaptic plasticity induced by neuronal excitation. In this study, we tested this hypothesis by studying relative gephyrin dynamics and GABA_A_R declustering during excitatory activity. Surprisingly, we found that gephyrin dispersal is not essential for GABA_A_R declustering during excitatory activity. In cultured hippocampal neurons, quantitative immunocytochemistry showed that the dispersal of synaptic GABA_A_Rs accompanied with neuronal excitation evoked by 4-aminopyridine (4AP) or *N*-methyl-D-aspartic acid (NMDA) precedes that of gephyrin. Single-particle tracking of quantum dot labeled-GABA_A_Rs revealed that excitation-induced enhancement of GABA_A_R lateral mobility also occurred before the shrinkage of gephyrin clusters. Physical inhibition of GABA_A_R lateral diffusion on the cell surface and inhibition of a Ca^2+^ dependent phosphatase, calcineurin, completely eliminated the 4AP-induced decrease in gephyrin cluster size, but not the NMDA-induced decrease in cluster size, suggesting the existence of two different mechanisms of gephyrin declustering during activity-dependent plasticity, a GABA_A_R-dependent regulatory mechanism and a GABA_A_R-independent one. Our results also indicate that GABA_A_R mobility and clustering after sustained excitatory activity is independent of gephyrin.

## Introduction

Inhibitory neurotransmission plays a critical role in the regulation of neuronal excitability and information processing in the brain. GABA-A receptors (GABA_A_Rs) are neurotransmitter receptors that mediate fast inhibitory neurotransmission in the central nervous system [1]. The number of GABA_A_Rs at the synapse is a factor that controls the efficacy of GABAergic transmission [2], [3]. The number of synaptic GABA_A_Rs can be altered within a few minutes depending on neuronal inputs in the hippocampus. A brief application of *N*-methyl-D-aspartic acid (NMDA), which induces a chemical form of long-term depression at excitatory synapses, results in elevated inhibitory synaptic transmission through the increase of surface GABA_A_R expression and synaptic accumulation of GABA_A_Rs [4], [5]. By contrast, the decrease in the number of functional postsynaptic GABA_A_Rs and GABAergic synaptic currents is induced by brief high-frequency stimulation of Schaffer collateral fibers that produce long-term potentiation of excitatory synaptic transmission or induction of status epilepticus [6], [7], [8], [9], [10]. The latter process, i.e., activity-dependent reduction in the number of synaptic GABA_A_Rs, is mediated by the increase in intracellular Ca^2+^ concentration followed by the activation of a Ca^2+^/calmodulin-activated phosphatase, calcineurin [7], [10]. Several lines of evidence have indicated that calcineurin modulates the number of synaptic GABA_A_Rs by regulating their lateral mobility through the dephosphorylation of Ser327 in the GABA_A_R γ2 subunit [11], [12], [13]. However, the detailed molecular mechanism underlying the activity-dependent change in postsynaptic GABA_A_R number remains unclear.

The interaction between neurotransmitter receptors and postsynaptic density proteins is an important factor that determines synaptic receptor number and density [14], [15]. Gephyrin is a scaffold protein that directly binds to the α1–α3 subunit of GABA_A_Rs [16], [17], [18] and multiple proteins including tubulin, forming clusters at the GABAergic synapse [19]. Gephyrin plays a critical role in the regulation of synaptic GABA_A_R stability because gene knockout, RNAi knockdown, and prevention of GABA_A_R–gephyrin interaction result in a decrease in the number and density of synaptic GABA_A_Rs and an increase in GABA_A_R mobility on the cell surface [16], [20], [21]. On the other hand, the formation and maintenance of synaptic gephyrin clusters also require synaptic localization of GABA_A_Rs [13], [22], [23], [24], [25], [26], [27]. A previous study revealed that the amount of postsynaptic gephyrin decreases when the number of synaptic GABA_A_Rs decreases as a result of excitatory activity [11]. In the present study, we tested the hypothesis that gephyrin declustering could be the starting point of this activity-induced regulation of GABA_A_R lateral mobility and the number of postsynaptic GABA_A_Rs. Contrary to this hypothesis, we found evidence suggesting that excitatory activity impacts clustering of GABA_A_Rs first and gephyrin later.

## Results

### Activity-dependent decrease in synaptic GABA_A_Rs precedes that in gephyrin

We have previously shown the decrease in synaptic GABA_A_Rs and gephyrin when excitatory activity is increased [11]. To examine the timing of this process, we tracked changes in the immunofluorescence of synaptic GABA_A_R, gephyrin, and the presynaptic marker protein synapsin after pharmacological neuronal stimulation every 2.5 min in cultured rat hippocampal neurons. For GABA_A_R labeling, we developed a custom-made antibody that recognizes the extracellular domain of rat GABA_A_R (amino acids 39–67). We confirmed that this antibody specifically recognized mouse GABA_A_R γ2 subunits expressed in HeLa cells (Fig. S1A–C). The antibody labeled clusters on the dendrites and cell bodies of cultured hippocampal neurons (Fig. S1D), as visualized by immunocytochemical staining with the antibody against the GABA_A_R γ2 subunit (amino acids 39–53) used in a previous study [11] (Fig. S1E). We therefore concluded that the anti-GABA_A_R γ2 antibody selectively recognizes the rodent GABA_A_R γ2 subunit.

**Figure 1:**
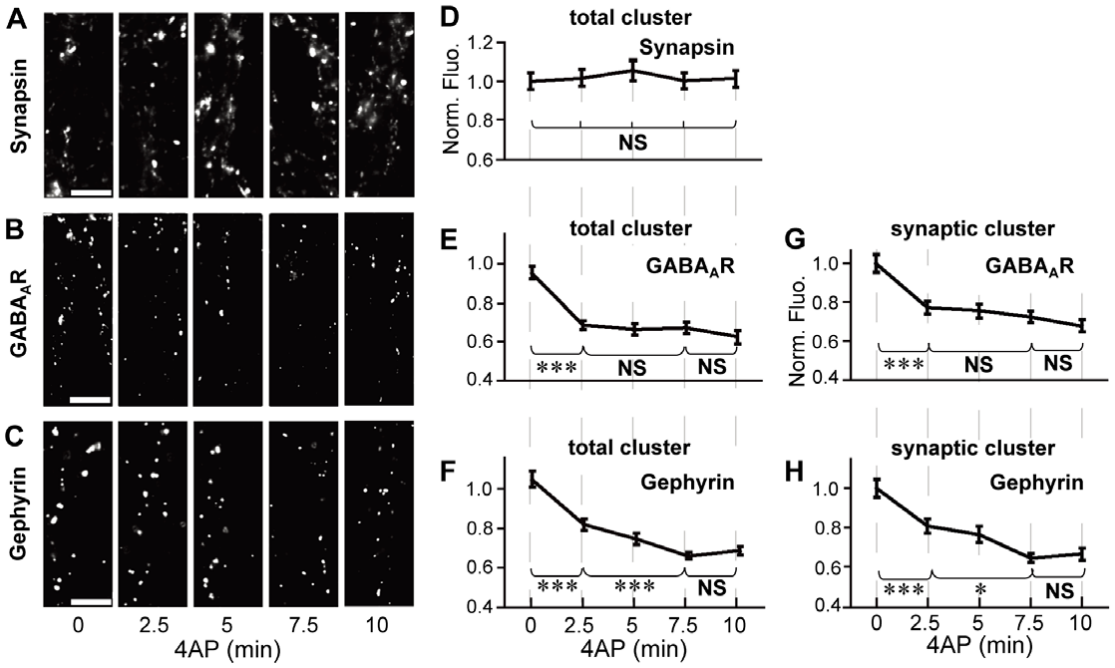
Time-course analysis of 4AP-induced decrease in GABA_A_R- and gephyrin-associated immunofluorescence. **A**–**C**: Representative examples of immunoreactivity associated with synapsin (**A**), GABA_A_R (**B**), and gephyrin (**C**) on the dendrites of hippocampal neurons (21–27 DIV) treated with 50 µM 4AP for 0–10 min. Scale bars: 5 µm. **D**–**F**: Time-course plots of changes in normalized fluorescence intensities (averages ± SEM) of total clusters of synapsin (**D**), GABA_A_R (**E**), and gephyrin (**F**) following 4AP treatment. **G**, **H**: Time-course plots of 4AP-induced reduction in the normalized fluorescence intensities of synaptic GABA_A_R (**G**) and gephyrin (**H**) clusters. NS: *p*>0.05, *: *p*<0.05, ***: *p*<0.005, Tukey's range test in ANOVA, n = 40 cells/condition (4 cultures).

Excitatory neuronal activity was induced by incubating cells with the potassium channel blocker 4-aminopyridine (4AP; 50 µM) for 2.5, 5, 7.5, and 10 min before fixation. Treatment with 4AP did not affect the immunofluorescent intensity of synapsin (Fig. 1A and D), suggesting that the increase in neuronal activity has only a minor effect on the size of presynaptic terminals. By contrast, the immunoreactivity associated with total (synaptic and extrasynaptic) GABA_A_Rs significantly decreased to 75.3%±2.3% of non-treated control cells within 2.5 min of incubation with 4AP (0 vs. 2.5 min, *p*<0.005, ANOVA; *p*<0.005, Tukey's range test in ANOVA), and no further decrease was induced by longer incubation (2.5–10 min, *p*>0.05, Tukey's range test in ANOVA; Fig. 1B and E). Total gephyrin immunoreactivity also decreased to 78.5%±2.7% of control cells (0 vs. 2.5 min, *p*<0.005, Tukey's range test in ANOVA) within 2.5 min. However, we observed a further decrease of gephyrin immunoreactivity to 63.9%±1.6% after incubation with 4AP for 7.5 min (2.5 vs. 7.5 min, *p*<0.005, Tukey's range test in ANOVA; Fig. 1C and F). Synaptic GABA_A_R and gephyrin clusters exhibited a time course similar to that of total GABA_A_R and gephyrin clusters (Fig. 1G and H). These results indicated that the activity-dependent decrease in the number of synaptic GABA_A_R clusters reached a steady state more quickly than that of synaptic gephyrin clusters.

Furthermore, we investigated the time courses of GABA_A_R- and gephyrin-associated immunofluorescence recovery after washout of 4AP (Fig. S2A–C). Both synaptic GABA_A_R and gephyrin immunoreactivity gradually recovered to almost the same level as that of non-treated cells within 10 min with a similar time course (Fig. S2E and F). No significant change in the size of the presynaptic terminals was detected by synapsin-associated immunofluorescence during the washout (Fig. S2D).

**Figure 2:**
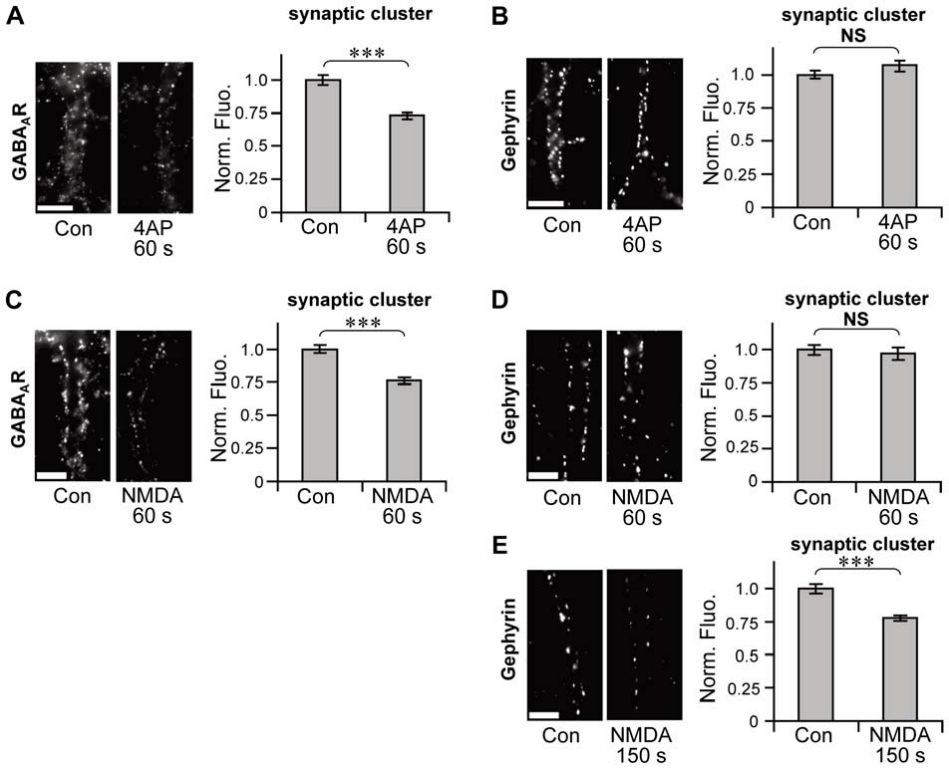
Activity-dependent decrease in synaptic clusters of GABA_A_R preceding that of gephyrin. Left: Representative examples of immunoreactivity associated with GABA_A_R (**A**, **C**) and gephyrin (**B**, **D**, **E**) in the presence (**A**, **B**: 4AP, **C**–**E**: NMDA) or absence (Con) of stimulation for the indicated times. Right: Normalized fluorescence intensities (averages ± SEM) of synaptic GABA_A_R (**A**, **C**) and gephyrin (**B**, **D**, **E**) clusters following stimulation. Note that fluorescence intensity of gephyrin was unchanged 60 s after the onset of stimulation (4AP: **B**, NMDA: **D**), while that of GABA_A_R significantly decreased at 60 s (4AP: **A**, NMDA: **C**). Scale bars: 5 µm. NS: *p*>0.05, ***: *p*<0.005, Welch's *t*-test, n = 30 cells/condition (3 cultures).

The comparison of the time courses of GABA_A_R and gephyrin clusters raised the possibility that the excitatory activity-induced reduction in GABA_A_R immunofluorescence precedes that in gephyrin immunofluorescence. Therefore, we further examined the 4AP-induced changes in GABA_A_R- and gephyrin-associated immunoreactivity within 2.5 min (150 s). Stimulation by 4AP for 60 s induced the reduction in synaptic GABA_A_R immunoreactivity to 73.0%±2.5% of control cells (*p*<0.005, Welch's *t*-test; Fig. 2A). However, synaptic gephyrin-associated immunofluorescence in the cells stimulated by 4AP for 60 s maintained the same intensity as observed in 4AP non-treated cells (106.6%±3.8% of control cells, *p*>0.05, Welch's *t*-test; Fig. 2B). We then examined the timing of NMDA-induced changes in GABA_A_R- and gephyrin-associated immunoreactivities, as the activation of the NMDA receptor and subsequent Ca^2+^ influx is also involved in the neuronal excitatory activity-dependent decrease in GABAergic synaptic transmission and declustering of GABA_A_Rs at inhibitory synapses [7], [12], [28]. When neurons were stimulated by 50 µM NMDA with its co-agonist, glycine, and TTX for 60 s, synaptic GABA_A_R immunoreactivity declined to 76.1%±2.3% of control cells (*p*<0.005, Welch's *t*-test; Fig. 2C). By contrast, synaptic gephyrin-associated immunofluorescence was unaffected by NMDA stimulation for 60 s (96.8%±4.7% of control cells, *p*>0.05, Welch's *t*-test; Fig. 2D). Longer NMDA treatment (150 s) resulted in the reduction of synaptic gephyrin immunoreactivity, as similarly observed with 4AP treatment; synaptic gephyrin immunoreactivity was reduced to 77.9%±2.2% of control cells (*p*<0.005, Welch's *t*-test; Fig. 2E). These results, together with the results of the time-course analysis of 4AP treatment, indicate that the excitatory activity-induced decrease in the number of synaptic GABA_A_Rs at postsynapses takes place before the shrinkage of synaptic gephyrin clusters.

### Modulation of GABA_A_R diffusion is complete before that of gephyrin clustering

**Figure 3:**
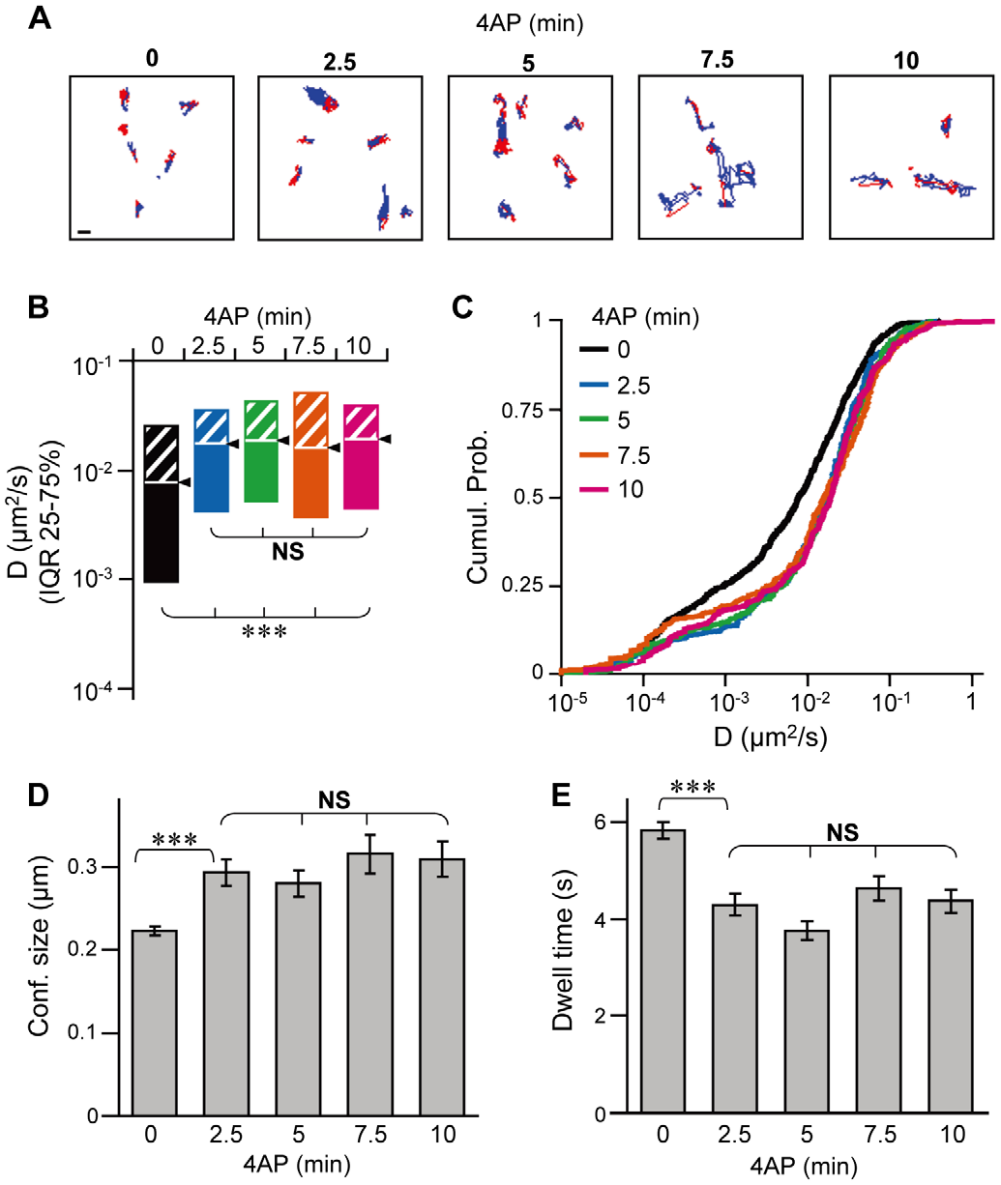
Modulation of GABA_A_R lateral diffusion by 4AP completed within 2.5 min. **A**: Examples of trajectories of GABA_A_R-QDs recorded for 38.9 s in neurons treated for the indicated times with 4AP, inside (red) and outside (blue) the synapse. Scale bar: 1 µm. **B**, **C**: The diffusion coefficients (D) of GABA_A_R-QDs inside the synapses in neurons incubated with 4AP for 0–2.5 min (2.5 min), 2.5–5 min (5 min), 5–7.5 min (7.5 min), and 7.5–10 min (10 min) and that in control cells (0 min). Median diffusion coefficients (triangle) and plus (hatched) and minus (solid) interquartile ranges (IQR) (**B**) and cumulative probability (**C**) of the synaptic diffusion coefficients of GABA_A_R-QDs. 0 min: n = 974 QDs; 2.5 min: n = 290; 5 min: n = 295; 7.5 min: n = 278; 10 min: n = 258. ***: *p*<0.005, NS: *p*>0.05, Kruskal–Wallis test. **D**: The average (±SEM) confinement sizes of GABA_A_R-QDs in synapses. 0 min: n = 500 QDs; 2.5 min: n = 122; 5 min: n = 106; 7.5 min: n = 127; 10 min: n = 111. **E**: The average synaptic dwell times (±SEM) of GABA_A_R-QD. 0 min: n = 3466 events; 2.5 min: n = 1147; 5 min: n = 1320; 7.5 min: n = 1131; 10 min: n = 1098. NS: *p*>0.05, ***: *p*<0.005, Tukey's range test in ANOVA (**D**, **E**). Data were obtained from 3 or 4 cultures.

The increase in GABA_A_R lateral diffusion dynamics plays a key role in neuronal activity-dependent decrease in GABA_A_R clustering at inhibitory synapses [11], [12]. Therefore, we conducted a time-course analysis of GABA_A_R lateral diffusion dynamics after 4AP stimulation using single-particle tracking with quantum dots (QD-SPT) [29]. Endogenous GABA_A_Rs were targeted with an antibody against the extracellular domain of the γ2 subunit (Fig. S1D) and subsequently labeled with an intermediate biotinylated Fab fragment and streptavidin-coated QDs. The lateral diffusion parameters after 4AP stimulation were calculated from the trajectories of GABA_A_Rs labeled with QDs (GABA_A_R-QDs) (Fig. 3A). The location of the active synapse was visualized by labeling with the amphiphilic dye FM4–64, induced after a burst of activity with 40 mM KCl. We confirmed that this FM4–64 labeling did not affect the GABA_A_R diffusion coefficient both in the absence and presence of 4AP treatment (Fig. S3A). The diffusion coefficient of GABA_A_R-QD at the synapse obtained by synaptic trajectories (red in Fig. 3A) was significantly increased within 2.5 min after the onset of 4AP stimulation (0–10 min, *p*<0.005, Kruskal–Wallis test; 0 vs. 2.5 min, *p*<0.005, Mann–Whitney *U* test; Fig. 3B and C). An additional increase in diffusion coefficient was not induced by longer incubation (2.5–10 min, *p*>0.05, Kruskal–Wallis test). In the absence of FM4–64 labeling, a 4AP-induced increase in diffusion coefficient was observed within 4 min (Fig. S3B), suggesting that the KCl-induced burst during FM4–64 labeling does not significantly impact the time course of 4AP-induced changes in the GABA_A_R diffusion coefficient. Forty to fifty percent of synaptic GABA_A_R-QD exhibited “confined diffusion,” i.e., lateral diffusion limited to a small surface area [30], as reported previously [11]. The size of confinement was calculated for this population (see Materials and Methods). The average confinement size was significantly increased to 131.4%±6.9% of control cells by 4AP treatment for 2.5 min (0 vs. 2.5 min, *p*<0.005, Tukey's range test in ANOVA) and then maintained during further incubation (2.5–10 min, *p*>0.05, Tukey's range test in ANOVA; Fig. 3D). Furthermore, the synaptic dwell time of GABA_A_R-QD decreased to 73.8%±3.8% of control cells at 2.5 min (0 vs. 2.5 min, *p*<0.005, Tukey's range test in ANOVA); however, no further decrease was observed after 2.5 min (2.5–10 min, *p*>0.05, Tukey's range test in ANOVA) (Fig. 3E). These results indicate that 4AP-dependent modification of GABA_A_R lateral diffusion reaches a steady state within 2.5 min, which probably leads to the decrease in the number of synaptic GABA_A_Rs (Fig. 1G). The time taken by gephyrin-associated immunofluorescence to reach a steady state was 7.5 min (Fig. 1H). This is 5 min longer than the time taken for GABA_A_R diffusion dynamics to reach a steady state. Therefore, our results indicate that the activity-dependent change in the lateral diffusion of GABA_A_Rs is completed before the dispersion of gephyrin clusters.

### 4AP-dependent modulation of gephyrin clusters depends on GABA_A_R lateral mobility

**Figure 4:**
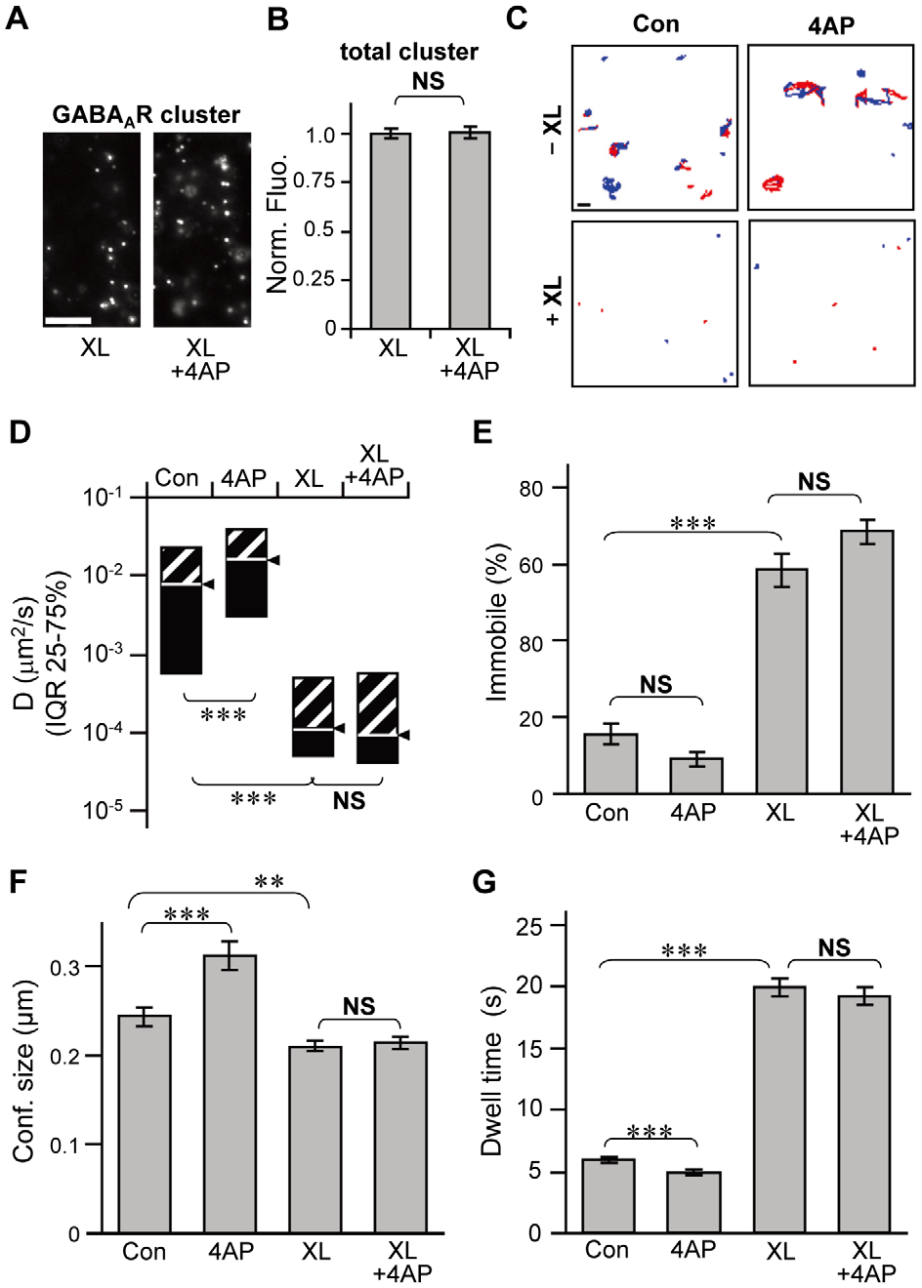
Inhibition of GABA_A_R lateral diffusion by cross-linking (XL). **A**: Examples of cross-linked GABA_A_R clusters on the dendrites of hippocampal neurons with (XL+4AP) or without (XL) 4AP stimulation. Scale bar: 5 µm. **B**: Normalized average fluorescent intensities of cross-linked GABA_A_R clusters with (XL+4AP) or without (XL) 4AP stimulation (averages ± SEM). XL: n = 39 cells; XL+4AP: n = 40 cells, from 4 cultures. **C**: Representative trajectories of GABA_A_R-QDs recorded for 38.9 s with (+XL) or without (−XL) XL in the presence or absence of 4AP, inside (red) and outside (blue) the synapse. Scale bar: 1 µm. **D**–**G**: Effects of XL and 4AP treatment on diffusion coefficients (**D**), percentage of immobile receptors (**E**), synaptic confinement sizes (**F**), and synaptic dwell times (**G**) of GABA_A_R-QDs. **D**: The diffusion coefficients of GABA_A_R-QDs (median ± IQR). The number of GABA_A_R-QDs analyzed: Con: n = 556; 4AP: n = 418; XL: n = 321; XL+4AP: n = 321. **E**: The percentage of immobile GABA_A_R-QDs (average ± SEM). Con: n = 15 cells; 4AP: n = 14; XL: n = 14; XL+4AP: n = 19. **F**: The confinement size (average ± SEM) of synaptic GABA_A_R-QDs. Con: n = 366 QDs; 4AP: n = 203; XL: n = 200; XL+4AP: n = 196. **G**: The average dwell times (±SEM) of GABA_A_R-QDs in synapses. Con: n = 2039 events; 4AP: n = 1576; XL: n = 473; XL+4AP: n = 493. Data in **D**–**G** were obtained from 3 cultures. NS: *p*>0.05, **: *p*<0.01, ***: *p*<0.005, Welch's *t*-test for **B**, **E**–**G**, and Mann–Whitney *U* test for **D**.

It is well established that synaptic gephyrin clustering also requires synaptic localization of GABA_A_Rs [13], [22], [23], [24], [25], [26], [27]. Based on the finding that the excitatory activity-induced modulation of GABA_A_R lateral diffusion was accomplished before gephyrin declustering, we hypothesized that gephyrin clustering could be sensitive to GABA_A_R diffusion dynamics, in addition to its existence and localization. To confirm this hypothesis, we manipulated GABA_A_R diffusion dynamics by artificially cross-linking (XL) the GABA_A_R γ2 subunits using antibodies, as performed previously for AMPA receptors and metabotropic glutamate receptors [31], [32]. Successful XL of GABA_A_Rs was confirmed by the appearance of fluorescent clusters labeled with the Alexa Fluor®-conjugated antibody used for XL of primary antibodies targeted to GABA_A_Rs (Fig. 4A). The fluorescence intensities of these cross-linked GABA_A_R clusters were not affected by 4AP treatment (Fig. 4B). Next GABA_A_R mobility was examined by QD-SPT. Trajectories revealed that the area explored by GABA_A_R-QDs were greatly reduced when surface GABA_A_Rs were cross-linked, both inside (red, Fig. 4C) and outside (blue, Fig. 4C) the synapses. In the absence of 4AP, XL induced an approximately 100-fold reduction in GABA_A_R-QD diffusion coefficients (Fig. 4D), an approximately 3.7-fold increase in the percentage of immobilized GABA_A_R-QD (Fig. 4E), a 13.6% decrease in the confinement size (Fig. 4F), and an approximately 3.4-fold increase in the synaptic dwell time (Fig. 4G), indicating that GABA_A_R-QD lateral diffusion is greatly inhibited by XL. Moreover, XL blocked the 4AP-induced significant increase in the diffusion coefficient, enlargement of confinement size, and decrease in the synaptic dwell time of GABA_A_R-QDs (Fig. 4D–G).

**Figure 5:**
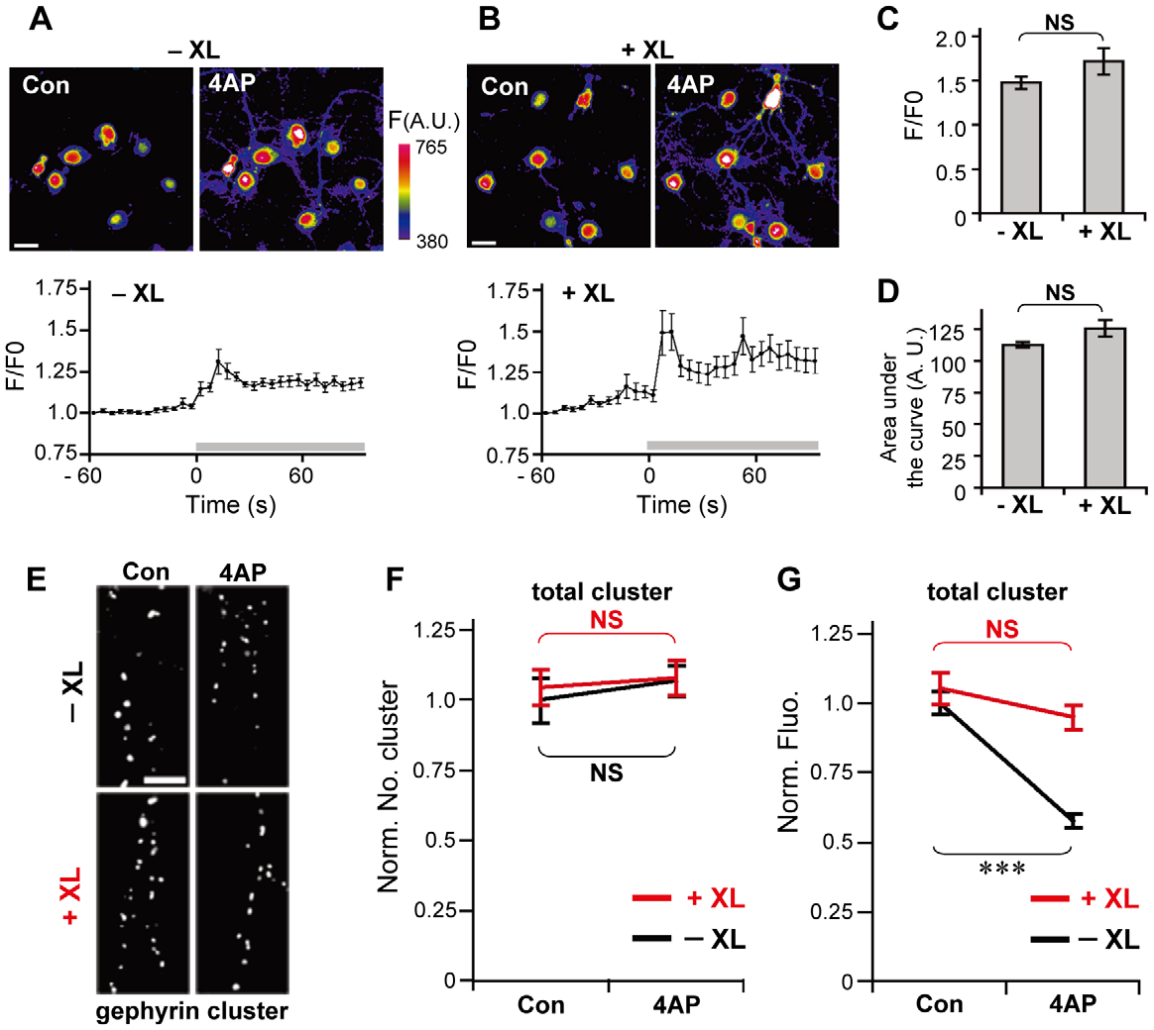
Suppression of 4AP-induced reduction in gephyrin immunofluorescence by GABA_A_R immobilization under increased levels of cytosolic Ca^2+^. **A**, **B**: Top: Representative pseudocolor images of hippocampal neurons loaded with fluo-4 AM without (**A**) or with (**B**) surface GABA_A_R XL, before (Con) and 10 s after 4AP application (4AP). Scale bars: 20 µm. Bottom: Time-course plots of F/F0 ratio changes (averages ± SEM) measured on proximal dendrites with the addition of 4AP, in the absence (**A**) or presence (**B**) of GABA_A_R XL. 4AP was applied at time = 0 as indicated by the gray horizontal bar in the traces. **C**, **D**: Peak amplitudes (**C**) and areas under the curve (**D**) for the F/F0-time plot during 90 s after addition of 4AP. Values indicate averages ± SEM. NS: *p*>0.05, Welch's *t*-test. −XL: n = 30 cells, +XL: n = 28, from 3 cultures. Note that normal increase in Ca^2+^ was induced by 4AP even under XL conditions. **E**: Examples of gephyrin-immunoreactive clusters in dendrites with (+XL) or without (−XL) surface GABA_A_R XL in the presence (4AP) and absence (Con) of 4AP treatment for 10 min. Scale bar, 5 µm. **F**, **G**: Effects of GABA_A_R XL and 4AP treatment on the normalized number of clusters (**F**) and normalized fluorescence intensities (**G**) of gephyrin clusters (averages ± SEM). NS: *p*>0.05; ***: *p*<0.005, Welch's *t*-test, n = 30 cells/condition (3 cultures). 4AP-induced reduction in gephyrin cluster size was completely suppressed by GABA_A_R XL.

We also confirmed that 4AP-induced increase in intracellular Ca^2+^ remained unaffected under XL conditions, which is responsible for the increase in GABA_A_R lateral diffusion. Ca^2+^ imaging with fluo-4 at proximal dendrites revealed that increase in intracellular Ca^2+^ was successfully induced by 4AP treatment even under XL conditions (Fig. 5B) as observed in the absence of XL (Fig. 5A), and that there was no significant difference in the peak amplitudes (Fig. 5C) and levels of increase in intracellular Ca^2+^ as represented by the area under the curve (Fig. 5D) between control and XL cells. Taken together, these experiments indicate that XL could inhibit GABA_A_R lateral diffusion without affecting intracellular Ca^2+^ elevation. Next we examined 4AP-induced declustering of gephyrin under XL conditions (Fig. 5E). Although a previous study showed that a 12-h XL of GABA_A_R resulted in the formation of extrasynaptic gephyrin clusters [33], the total number of gephyrin clusters in GABA_A_R XL conditions was not different from that without XL (Fig. 5F), suggesting that extrasynaptic artificial gephyrin clusters are not formed under our XL conditions. In the cells without GABA_A_R XL, 4AP incubation for 15 min significantly decreased gephyrin-associated immunoreactivity [Fig. 5G (−XL)]. Conversely, the same 4AP stimulation failed to induce reduction in gephyrin immunofluorescence in the cells with GABA_A_R XL [Fig. 5G (+XL)).

**Figure 6:**
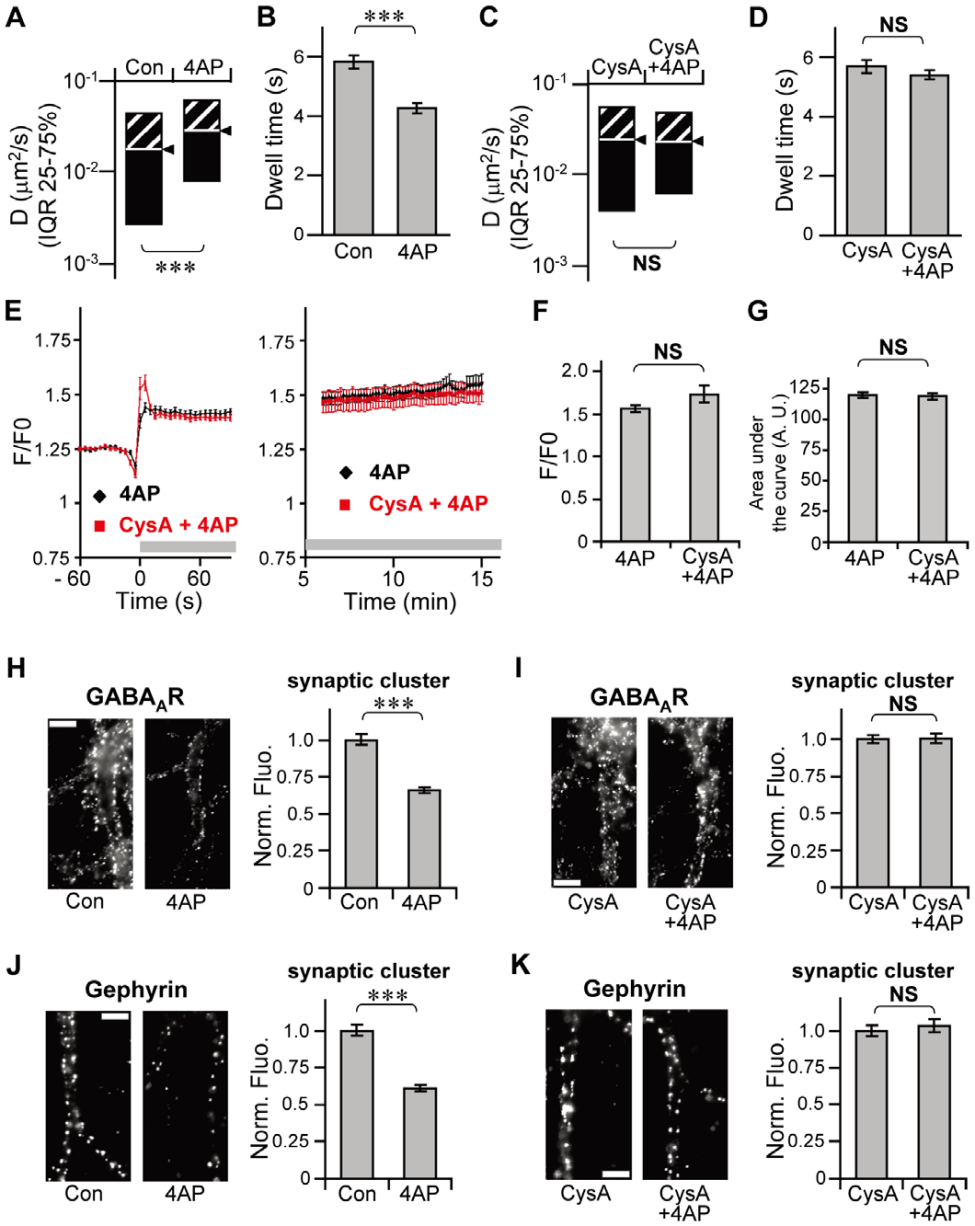
Prevention of 4AP-induced gephyrin declustering by calcineurin inhibitor CysA. **A**–**D**: Inhibition of 4AP-driven mobilization of GABA_A_R-QDs by CysA treatment. Diffusion coefficients in the synapse (**A** and **C**, median ± IQR) and synaptic dwell times (**B** and **D**, averages ± SEM) in the absence (**A**, **B**) or presence (**C**, **D**) of 1 µM CysA. NS: *p*>0.05, ***: *p*<0.005, Mann–Whitney *U* test for **A**, **C** (Con: n = 535 QDs, 4AP: n = 537, CysA: n = 478, CysA+4AP: n = 506.), Welch's *t*-test for **B**, **D** (Con: n = 2107 events, 4AP: n = 2505, CysA: n = 1930, CysA+4AP: n = 2094). Data were obtained from 3 cultures. **E**–**G**: Intact 4AP-induced increase in cytosolic Ca^2+^ concentration under CysA treatment. Changes in intracellular Ca^2+^ levels indicated as F/F0 of fluo-4 after the addition of 4AP (black) or CysA+4AP (red) (**E**). Drugs, i.e. 4AP or CysA+4AP, were applied at time = 0 as indicated by the gray horizontal bar in the traces. Peak amplitudes of F/F0 (**F**) and areas under the F/F0-time curve (**G**) during 90 s after the onset of stimulation. Values indicate averages ± SEM. NS: *p*>0.05, Welch's *t*-test. 4AP: n = 26 cells, CysA+4AP: n = 28 cells (3 cultures). **H**–**K**: The effect of CysA treatment on 4AP-driven declustering of GABA_A_R and gephyrin. Left: Example of immunoreactivity associated with GABA_A_R (**H**, **I**) and gephyrin (**J**, **K**) on the dendrites treated with 4AP for 30 min, in the absence (**H**, **J**) and presence (**I**, **K**) of CysA. Scale bars: 5 µm. Right: Normalized fluorescence intensities (averages ± SEM) of synaptic GABA_A_R (**H**, **I**) and gephyrin (**J**, **K**) clusters. NS: *p*>0.05, ***: *p*<0.005, Welch's *t*-test. Con, CysA: n = 30 cells/condition, 4AP, CysA+4AP: n = 25 cells/condition from 3 cultures.

XL of surface GABA_A_Rs is an extreme condition in which a large proportion of surface GABA_A_Rs are immobilized. Therefore, we also examined the effect of a calcineurin inhibitor, cyclosporin A (CysA), which does not immobilize surface GABA_A_Rs but suppresses the NMDA-induced increase in GABA_A_R mobility [11], [12], on gephyrin clustering. We confirmed that the 4AP-driven increase in the synaptic diffusion coefficient (Fig. 6A) and reduction in the synaptic dwell time (Fig. 6B) were completely inhibited in the presence of 1 µM CysA (Fig. 6C and D), which is in agreement with previous studies of NMDA stimulation [11], [12]. Ca^2+^ imaging with fluo-4 revealed that increase in intracellular Ca^2+^, sustained for at least 15 min, was normally induced by 4AP even in the presence of CysA (Fig. 6E). The peak amplitude (Fig. 6F) and Ca^2+^ influx level represented by the area under the curve (Fig. 6G) was not significantly affected by CysA (p>0.05, Welch's *t*-test). Under this condition, the size of synaptic clusters of GABA_A_R and gephyrin was quantified by immunocytochemistry. The shrinkage of synaptic GABA_A_R clusters induced by 4AP stimulation for 30 min (Fig. 6H) was blocked completely in the presence of CysA (Fig. 6I). Furthermore, 4AP-driven gephyrin declustering at the synapse (Fig. 6J) was also prevented by CysA treatment (Fig. 6K), despite the increase in cytosolic Ca^2+^.

In summary, the above results indicate that 4AP-driven gephyrin declustering is inhibited when there is no increase in GABA_A_R lateral diffusion in response to neuronal excitation. Our results also imply that synaptic gephyrin clustering is dependent on the mobility of GABA_A_Rs during sustained activity induced by 4AP.

### NMDA-driven gephyrin declustering is independent of GABA_A_R mobility

**Figure 7:**
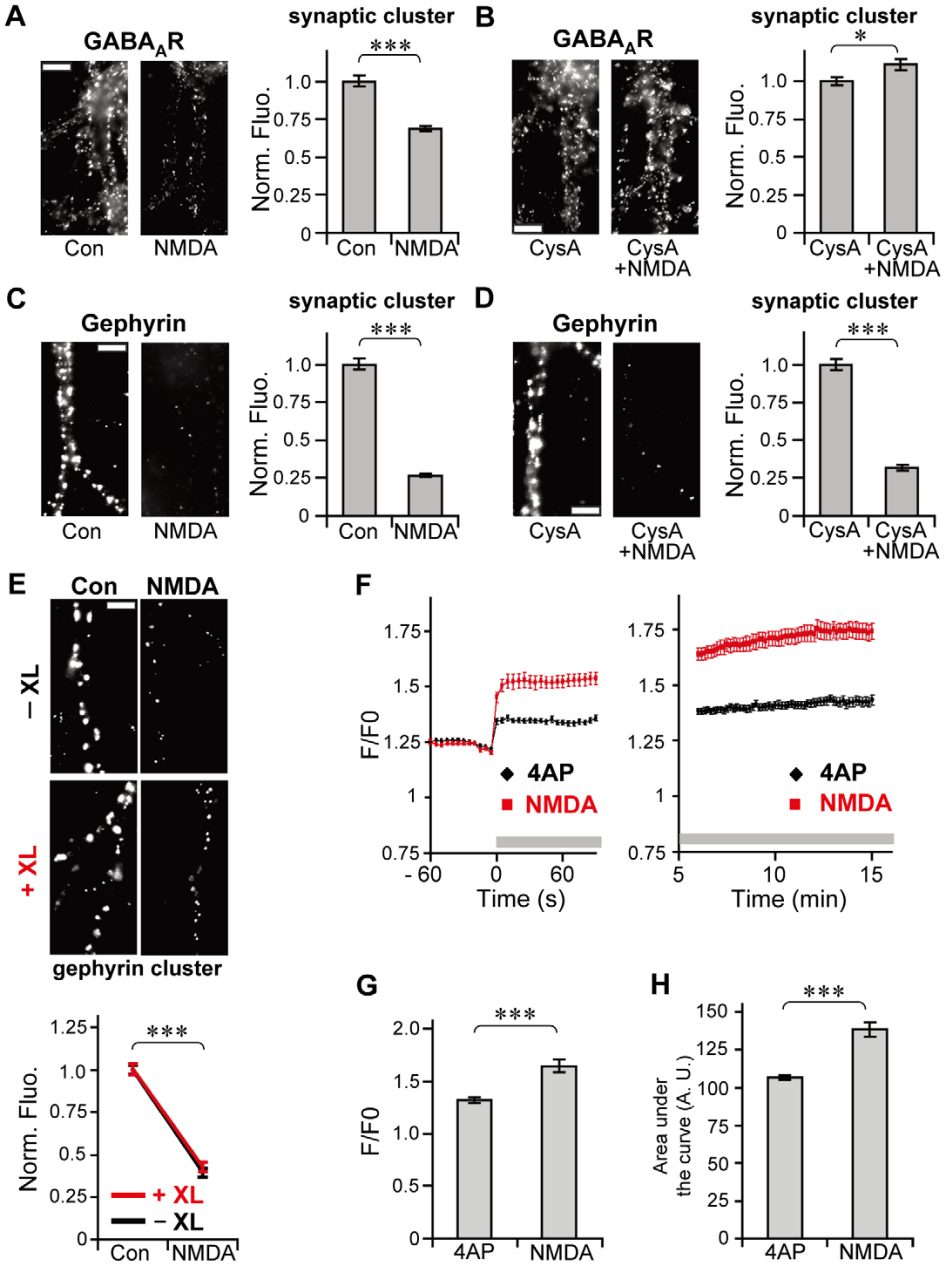
GABA_A_R-independent gephyrin declustering during sustained activity induced by NMDA stimulation. **A**–**D**: Effect of CysA treatment on NMDA-driven dispersal of GABA_A_R and gephyrin clusters. Left: Examples of GABA_A_R (**A**, **B**) and gephyrin (**C**, **D**) immunoreactivity in neurons incubated with NMDA for 30 min, with (**B**, **D**) and without CysA (**A**, **C**). Scale bars, 5 µm. Right: Normalized fluorescence intensities (averages ± SEM) of synaptic GABA_A_R (**A**, **B**) and gephyrin (**C**, **D**) clusters. *: *p*<0.05, ***: *p*<0.005, Welch's *t*-test. Con, CysA, CysA+NMDA: n = 30 cells/condition, NMDA: n = 25 cells/condition (3 cultures). CysA suppressed NMDA-induced dispersal of GABA_A_R clusters, but not that of gephyrin clusters. **E**: NMDA-induced gephyrin dispersal under GABA_A_R XL. Top: Gephyrin immunoreactive clusters in neurons with (+XL) and without (−XL) surface GABA_A_R XL after NMDA stimulation. Bottom: Effects of GABA_A_R XL and NMDA treatment on the normalized fluorescence intensity of gephyrin clusters (average ± SEM). ***: *p*<0.005, Welch's *t*-test, n = 30 cells/condition (3 cultures). **F**–**H**: Comparison of the Ca^2+^ influx level induced by 4AP and NMDA. Increase in Ca^2+^ after the addition of 4AP (black) or NMDA (red) (**F**). Gray horizontal bars in the traces indicate the presence of 4AP or NMDA. Peak amplitudes (**G**) and areas under the curve (**H**) for F/F0-time plots during 90 s after the onset of stimulation. Values indicate averages ± SEM. ***: *p*<0.005, Welch's *t*-test. 4AP: n = 21 cells, NMDA: n = 23 cells (3 cultures).

The result of GABA_A_R XL and CysA experiments with 4AP stimulation suggested the existence of a mechanism, dependent on GABA_A_R surface mobility, which regulates gephyrin clustering. Finally, we examined whether gephyrin clustering is constantly subjected to this GABA_A_R-dependent regulation during sustained neuronal excitation. NMDA stimulation was applied to increase neuronal activity, and effects of CysA treatment on synaptic GABA_A_R and gephyrin clusters were examined. In agreement with previous reports that CysA inhibits NMDA-induced increase in GABA_A_R lateral diffusion [11], [12] and declustering of GABA_A_Rs [12], the dispersal of synaptic GABA_A_R observed after 30 min of NMDA treatment (Fig. 7A) was completely blocked by the presence of CysA (Fig. 7B). NMDA stimulation significantly diminished the size of gephyrin clusters to 26.7%±0.9% of control cells (Fig. 7C). Unlike the GABA_A_R clusters, synaptic gephyrin clusters were reduced (31.0%±2.1% of control cells, Fig. 7D) even in the presence of CysA. XL of surface GABA_A_Rs also failed to inhibit NMDA-induced declustering of gephyrin (Fig. 7E). Interestingly, increase in Ca^2+^ induced by NMDA stimulation, which persisted for at least 15 min, was larger than that induced by 4AP (Fig. 7F). The average peak amplitude of Ca^2+^ elevation evoked by NMDA was 1.2 times larger than that induced by 4AP (p<0.005, Welch's *t*-test; Fig. 7G) and the level of increase in Ca^2+^ during NMDA stimulation was 1.3 times higher than that during 4AP stimulation (p<0.005, Welch's *t*-test; Fig. 7H). Taken together, these results suggest that gephyrin clustering is not dependent on GABA_A_R mobility during sustained activity induced by NMDA, possibly at high levels of increase in Ca^2+^. More importantly, despite the loss of synaptic gephyrin clustering by NMDA stimulation (Fig. 7D), Cys A blocked NMDA-induced declustering of GABA_A_Rs (Fig. 7B) and the increase in lateral diffusion [11], [12]. These results clearly indicate that lateral diffusion of GABA_A_Rs at the synapse and synaptic GABA_A_R clustering during inhibitory synaptic plasticity are independent of the amount of synaptic gephyrin present.

## Discussion

The main finding of this study is that changes in lateral diffusion dynamics and number of synaptic GABA_A_Rs preceded gephyrin declustering during excitatory activity. In addition, our results indicate that synaptic GABA_A_R diffusion and clustering are independent of the status of gephyrin clusters during sustained excitatory activity.

Gephyrin is considered a key protein that controls GABA_A_R stability at the postsynapse [13], [16], [20], [21]. In this study, we tested the hypothesis that the excitatory activity-dependent reduction in postsynaptic GABA_A_Rs [11], [12], which could be involved in GABAergic synaptic plasticity, is initiated by the dispersion of gephyrin from clusters. If this hypothesis were correct, excitatory activity should have affected gephyrin first or at least at the same time when affecting GABA_A_Rs. Contrary to this expectation, a detailed time-course analysis indicated that the dispersal of GABA_A_R clusters induced by the enhancement of GABA_A_R lateral mobility preceded the dispersal of gephyrin. Our results suggest that neuronal activity-induced rapid decrease in GABA_A_R numbers at mature inhibitory synapses is not mediated by gephyrin declustering. This notion was further supported by the observation that synaptic GABA_A_R mobility and clustering were not affected by NMDA in the presence of CysA, while gephyrin cluster largely decreased under the same conditions. Our findings suggest that excitatory activity-induced plasticity in GABAergic synapses is induced independent of the status of gephyrin clusters.

There was no remarkable difference in the recovery time course of GABA_A_R and gephyrin cluster size after 4AP removal, similar to the process of synaptogenesis in hippocampal neurons [34], [35]. This suggests that the reaccumulation of GABA_A_R and gephyrin to the inhibitory synapse occurs simultaneously. It remains unclear whether gephyrin is critical for the recovery of GABA_A_R clusters.

Furthermore, our results suggested that there are existence two regulatory mechanisms of gephyrin clustering during sustained activity: GABA_A_R-dependent and GABA_A_R-independent mechanisms. The amount of gephyrin in clusters was maintained even in the presence of 4AP, when surface GABA_A_Rs were immobilized by XL and when 4AP-induced increase in GABA_A_R diffusion was prevented by CysA-treatment. This finding indicates that GABA_A_R lateral diffusion dynamics can affect clustering of the scaffold protein gephyrin. Recent theoretical modeling of postsynaptic structures based on chemical potential proposed another concept which states that the stabilization of the postsynaptic structure is reciprocal. In other words, scaffold proteins stabilize receptors and receptors stabilize scaffold proteins [36]. Together with the fact that gephyrin is crucial for the stabilization of postsynaptic GABA_A_Rs [16], [20], [21], our data provide direct evidence of a reciprocal mechanism that stabilizes the structure of GABAergic synapses. Regulation of postsynaptic scaffolds by neurotransmitter receptors is involved in synaptogenesis and the maintenance of GABAergic synapses, as evidenced by the fact that the absence of some GABA_A_R subunits results in the disappearance of gephyrin clusters [22], [23], [24], [25], [26], [27]. Our present results, which imply that activity-induced mobilization of surface GABA_A_Rs destabilizes gephyrin clusters, also raise the possibility that GABA_A_R lateral mobility, in addition to its existence and localization, could be a primary determinant of stability of mature GABAergic synaptic structures during synaptic plasticity. Changes in the chemical potential associated with GABA_A_Rs and gephyrin, which are induced by the enhancement of lateral diffusion and subsequent decrease in synaptic GABA_A_R density, could lead to a new steady state of postsynaptic molecular assembly [36].

The observation that gephyrin dispersed after NMDA stimulation regardless of GABA_A_R mobility suggested that another GABA_A_R-independent regulatory mechanism may control gephyrin clustering. Considering that NMDA induced a 1.3 times larger Ca^2+^ elevation than 4AP, the Ca^2+^ influx level could be one of the factors determining whether gephyrin is subjected to GABA_A_R-dependent regulation or independently destabilized in response to Ca^2+^ elevation. Gephyrin is a substrate of the Ca^2+^-dependent non-lysosomal cysteine protease calpain-1, which is activated when NMDA receptors are stimulated [37], and turnover of gephyrin is regulated by calpain-1 activity [38]. Therefore, it is possible that gephyrin stability is also controlled by the activation of calpain-1 during NMDA stimulation [39]. However, it must be noted that the same NMDA stimulation (50 µM, with glycine and TTX) did not induce gephyrin declustering in cultured spinal cord neurons [40], in which calpain-1 is also activated by NMDA stimulation [41]. Thus, the molecular mechanism for this GABA_A_R-independent gephyrin regulation remains to be elucidated by future studies.

Activity-dependent regulation of GABA_A_R lateral diffusion and clustering at inhibitory synapses is mediated by Ca^2+^ influx and subsequent activation of calcineurin [11], [12], [13]. Our present findings provide several insights into the molecular mechanism of how Ca^2+^ signaling enhances GABA_A_R lateral diffusion. In the present study, we found that GABA_A_R diffusion and clustering were independent of gephyrin clustering during NMDA stimulation in the presence of CysA. This finding strongly suggests that calcineurin-dependent regulation of GABA_A_R mobility does not require gephyrin. Because alterations in receptor–scaffold interactions can modulate the lateral diffusion of receptors [15], we propose the existence of other GABA_A_R-interacting protein(s) that contribute to GABA_A_R stabilization in a gephyrin-independent manner. GABA_A_R accumulation at the inhibitory synapse occurs before gephyrin accumulation during synaptogenesis in spinal cord neurons [42], suggesting the existence of a gephyrin-independent stabilization mechanism of GABA_A_Rs. This gephyrin-independent pathway may enhance GABA_A_R lateral diffusion via the calcineurin-dependent dephosphorylation of Ser327 in the GABA_A_R γ2 subunit [12]. We speculate that the dephosphorylation of Ser327 upon neuronal excitation induces the dissociation of unidentified GABA_A_R-associating protein(s) from GABA_A_Rs, which leads to the observed increase in GABA_A_R lateral mobility.

The Ca^2+^-dependent increase in GABA_A_R lateral mobility is involved in synaptic plasticity at inhibitory synapses that may underlie neuronal disorders resulting from pathological disinhibition [11], [12]. Therefore, elucidating the detailed molecular mechanism of the gephyrin-independent regulation of GABA_A_R lateral mobility might contribute not only to understanding the basis of learning and memory but also to discovering therapeutic targets for neuropathies such as epilepsy.

## Materials and Methods

### Ethics statement

All animal procedures in this study were performed in accordance with the guidelines issued by the Japanese Ministry of Education, Culture, Sports, Science and Technology. All animal procedures in this study were approved by the Animal Experiment Committee of the RIKEN (H23-2-204). All efforts were made to minimize animal suffering and reduce the number of animals used.

### Anti-GABA_A_R γ2 subunit antibody production

The rabbit anti-GABA_A_R γ2 subunit antibody (anti-GABA_A_Rγ2) was raised against the peptide “QKSDDDYEDYASNKTWVLTPKVPEGDVTV(C)” corresponding to amino acid residues 39–67 of the rat GABA_A_R γ2 subunit, as shown previously [43]. The peptide was synthesized by the Support Unit for Bio-material Analysis at the RIKEN BSI Research Resources Center (RRC) and was subsequently injected into rabbits to obtain the antibody by the Support Unit for Animal Resources Development at the RIKEN BSI RRC.

The specificity of the antibody was confirmed using HeLa cells (RIKEN BioResource Center, Ibaraki, Japan) expressing α1, β3, and γ2 subunits of GABA_A_R (Fig. 1A and C). HeLa cells were plated onto 18-mm diameter glass coverslips and cultured in DMEM (Nacalai Tesque, Kyoto, Japan) supplemented with 10% fetal bovine serum and antibiotics. For transfection, a coverslip in 1 ml culture medium was incubated with the transfection mixture containing 100 µl OPTI-MEM (Invitrogen, Tokyo, Japan), mixture of DNA (α1, β3, γ2; 0.7 µg each), and 4.2 µl TransIT-LT1 (Mirus, WI, USA) for 24 h before observation. Plasmids encoding α1, β3, and γ2 subunits of GABA_A_R were generated by subcloning the coding region into the mammalian expression vector [pcDNA3.1/Zeo(+/−); Invitrogen] using FANTOM3 clones as PCR templates (α1: C630037M06; β3: C630014N19; γ2: B930018F17 and C230063G02) [44].

### Primary cultures

Primary cultures of hippocampal neurons co-cultured with astrocytes were prepared from E18–21 Wistar rat embryos as previously described [45] with some modifications. Hippocampal cells were dissociated in plating medium comprising minimum essential medium (MEM; Invitrogen) supplemented with B27 (Invitrogen), 2 mM L-glutamine, 1 mM sodium pyruvate (Invitrogen), and antibiotics, and were plated at a density of 1.4×10^5^ cells/ml onto 18-mm diameter glass coverslips precoated with 0.04% polyethyleneimine (Sigma, Tokyo, Japan). Three days after plating, the culture medium was replaced with maintenance medium comprising Neurobasal-A medium (Invitrogen) supplemented with B27, 2 mM L-glutamine, and antibiotics. Cells were cultured for 21–27 days *in vitro* before the experiments. At least three independent cultures were used for each experiment.

### Drug treatment

To increase excitatory activity, cultured hippocampal neurons were incubated with 50 µM 4AP (Nacalai Tesque) or 50 µM NMDA (Tocris, MO, USA), glycine (5 µM), and TTX (1 µM; Tocris) at 37°C in the imaging medium comprising MEM without phenol red (Invitrogen), 20 mM HEPES, 33 mM glucose, 2 mM glutamine, 1 mM sodium pyruvate, and B27. For time-course analysis of cluster recovery, neurons were treated with 50 µM 4AP for 10 min and subsequently incubated with the imaging medium for 0–15 min before fixation. For QD-SPT experiments, 4AP (final concentration, 50 µM) was added to the imaging medium immediately before recording. For Ca^2+^ imaging, recording were done for 1 min in the absence of drugs, then drugs were bath applied to the cells during the recording.

### Immunocytochemistry and quantitative analysis

For GABA_A_R immunostaining of cultured neurons with drug treatment, endogenous GABA_A_Rs on cultured hippocampal neurons were labeled with our γ2 antibodies by incubating live cells with 2.0 µg/ml antibody diluted in imaging medium for 30 min at 37°C. Subsequently, cells were stimulated by 4AP or NMDA and fixed with 4% (w/v) paraformaldehyde (PFA) in PBS-0.02% NaN_3_ at room temperature (24–26°C) for 15 min. After permeabilization with 0.1% triton X-100 for 3 min and incubation with 5% (w/v) bovine serum albumin (BSA; Sigma) for 30 min to block nonspecific staining, cells were labeled with the mouse anti-synapsin I antibody (1∶3000; Synaptic Systems, Goettingen, Germany) in 2.5% BSA for 60 min. After washes, the cells were incubated in Alexa Fluor®-conjugated secondary antibodies (5–10 µg/ml, Alexa Fluor 488 or Alexa Fluor 594; Invitrogen) for 30 min, washed, and mounted on slides with Vectashield (Vector Laboratories, CA, USA). In the experiments using the calcineurin inhibitor CysA (1 µM; Santa Cruz Biotechnology, CA, USA), cells were incubated with our γ2 antibodies (2.0 µg/ml) for 30 min in the presence of drug (i.e., 4AP, NMDA+TTX+Gly, CysA) and subsequently fixed by 4% PFA. After fixation, the procedures were the same as those of experiments without CysA treatment. In some experiments (Fig. S1E), GABA_A_R was labeled with commercially available rabbit anti-γ2 subunit antibodies (6.0 µg/ml; Alomone Labs, Jerusalem, Israel), which were used in a previous study [11]. GABA_A_Rs on the GABA_A_R-expressing HeLa cells were labeled with our custom-made anti-GABA_A_Rγ2 antibody (0.8 µg/ml) as described above, and nuclei of HeLa cells were stained with DAPI.

For labeling of gephyrin, cells were fixed with 4% PFA after drug stimulation and permeabilized with 0.1% Triton X-100. After blocking with 5% BSA, cells were incubated with anti-gephyrin antibody (0.33 µg/ml, clone mAb7a; Synaptic Systems) and the rabbit polyclonal anti-synapsin I antibody (1∶400; Millipore, MA, USA) in the presence of 2.5% BSA for 90 min, and subsequently labeled with Alexa Fluor 488 or Alexa Fluor 594 (5–10 µg/ml; Invitrogen).

Immunofluorescence from isolated neurons was acquired on an inverted microscope (IX-70; Olympus, Tokyo, Japan) equipped with a Plan Apo 60× oil immersion objective with a numerical aperture (NA) of 1.42 (Olympus), cooled CCD camera (Orca-II-ER; Hamamatsu Photonics, Shizuoka, Japan), and appropriate filter sets for Alexa Fluor 488 (ex: 480±10 nm, em: 530±20 nm) and Alexa Fluor 594 (ex: 535±15 nm, em: 580 nm long pass). All images from a given culture were acquired with the same subsaturation exposure time.

Quantification of GABA_A_R-, gephyrin-, and synapsin-associated immunofluorescence was performed using “Integrated Morphometry Analysis” function of the MetaMorph software (Molecular Device Japan, Tokyo, Japan). GABA_A_R- and gephyrin-immunoreactive clusters and synapsin-positive presynapses were defined by processing images with multidimensional image analysis (MIA) interface, i.e., a 2D object segmentation by wavelet transform [46] and “auto threshold for light object (isodata method)” function of MetaMorph. Synaptic GABA_A_R or gephyrin clusters were defined as clusters that overlapped at least 1 pixel with presynaptic terminals. For each culture, all cluster fluorescence intensity was normalized to the average value in control cells.

### QD-SPT experiments

Neurons were incubated with the custom-made anti-GABA_A_Rγ2 antibody (2.0 µg/ml) for 5 min, washed, and incubated with the biotinylated anti-rabbit Fab antibody (2.2 µg/ml; Jackson ImmunoResearch, PA, USA) for 5 min. Following washes, the coverslips were incubated with 1.0 nM streptavidin-coated QDs emitting at 605 nm or 625 nm (Invitrogen) in borate buffer for 1 min [29]. After washes, functional presynaptic boutons were labeled with 2 µM FM4–64 (Invitrogen) in imaging medium containing 40 mM KCl for 15 s. Incubation with antibodies and washes were performed at 37°C in the imaging medium.

The diffusive behavior of GABA_A_R-QD and FM4–64 signals was recorded at 37°C in the imaging medium using an inverted microscope (IX-71, Olympus) equipped with an oil immersion objective (NA 1.45, 60×; Olympus) and an EM-CCD camera (C9100; Hamamatsu Photonics) or an inverted microscope (IX-70; Olympus) equipped with an oil immersion objective (NA 1.42, 60×; Olympus) and cooled CCD camera (Orca-II-ER; Hamamatsu Photonics). Fluorescent signals were detected using appropriate filter sets for QD (ex: 455±70 nm, em: 605±20 nm) and FM4–64 (ex: 535±15 nm, em: 580 nm long pass). GABA_A_R-QD lateral diffusion was recorded with an integration time of 76 ms with 512 consecutive frames (38.9 s). All recordings were taken within 30 min.

### Data analysis for QD-SPT experiments

The trajectory of GABA_A_R-QD was obtained by cross-correlating images with a Gaussian model of the point spread function [47], and diffusion coefficients and confinements were calculated using TI workbench software written by Dr. T. Inoue (Waseda University), as described previously [11]. Only single QDs identified by intermittent fluorescence (i.e., blinking) were analyzed. The synaptic area was defined by processing FM4–64 images with wavelet decomposition [46]. GABA_A_R-QDs were classified as “synaptic” when overlapping with synaptic area+2 pixels (284 nm). For the calculation of diffusion parameters in the synapse except for synaptic dwell time, the longest sub-trajectories of single GABA_A_R-QDs with greater than or equal to 30 points in each compartment were taken into account.

To obtain the diffusion parameters, such as the diffusion coefficient and confinement size, values of the mean square displacement (MSD) plot versus time were calculated for each trajectory by applying the following equation:
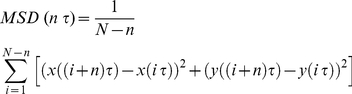
(1)([48]), where *τ* is the acquisition time, *N* is the total number of frames, and *n* and *i* are positive integers with *n* representing the time increment. Diffusion coefficients (*D*) were calculated by fitting first four points of the MSD versus time curves with the following equation:
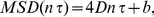
(2)where *b* is a constant reflecting the spot localization accuracy. In this system, GABA_A_R-QDs with a diffusion coefficient (D) less than 0.0002 µm^2^/s were defined as immobile.

The confinement domain size, in which the diffusion of GABA_A_R-QD was restricted, was obtained by fitting the MSD-nτ plot to the following equation:
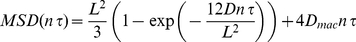
(3)
[30], where *L^2^* is the confined area in which diffusion is restricted, and *D_mac_* is the diffusion coefficient on a long time scale. The diffusion of GABA_A_R-QD with MSD-nτ plot that does not apply |D-D_mac_|<0.1×D or L<0.001 was defined as restricted motion, and only GABA_A_R-QDs meeting this criteria were considered for calculations of confinement domain sizes [49].

The GABA_A_R-QD dwell time inside the synapse was defined as the duration of synaptic sub-trajectories.

### GABA_A_R XL experiments

GABA_A_Rs on the cell surface were cross-linked by incubating neurons with the anti-γ2 subunit antibody (8.0 µg/ml; Alomone Labs) for 10 min, washing, and incubating with Alexa Fluor®-conjugated anti-rabbit antibodies (20 µg/ml; Invitrogen) for 5 min in the imaging medium. Cells were further incubated with the biotinylated anti-rabbit Fab antibody and streptavidin-coated QDs for QD-SPT, or fixed and subsequently immunolabeled with the gephyrin antibody for quantitative immunocytochemistry, as mentioned previously. In all experiments, it was confirmed that surface GABA_A_Rs were successfully cross-linked by fluorescence from GABA_A_R-associated clusters (Fig. 4A).

### Ca^2+^ imaging

Neurons were loaded with 0.5 µM fluo-4 AM (Invitrogen) for 5 min at 37°C. Fluo-4 fluorescence was acquired at 0.2 Hz with a 200-ms exposure at room temperature (24–26°C), with an inverted microscope (IX-70; Olympus) equipped with a 40× objective (NA 0.85, UPlanApo; Olympus), a cooled CCD camera (Orca-II-ER; Hamamatsu Photonics), and appropriate filters (ex, 480±10 nm; em, 530±20 nm). For longer recording (Figs. 6E and 7E), images were further acquired at 0.1 Hz from 6 min to 15 min after drug application. Data were analyzed using a TI Workbench. The ratio of the fluorescence intensities F/F0, where F is a fluorescence intensity and F0 is the intensity at t = 0, was calculated after subtraction of the background fluorescence. To estimate the level of Ca^2+^ elevation, the area under the curve was calculated using Igor Pro software (WaveMetrics, OR, USA).

### Statistical analysis and image preparation

Statistical differences of data in the time course were determined using the Kruskal–Wallis (for the diffusion coefficient) and one-way ANOVA (*p* = 0.05) tests, followed by Tukey's post-hoc tests (for others). For comparisons between two groups, the Mann–Whitney *U* test or Welch's *t*-test were performed as indicated. All statistical analysis was performed using KaleidaGraph (Synergy Software, PA, USA). Images were prepared for printing using MetaMorph, Adobe Photoshop, and Adobe Illustrator.

## Supporting Information

Figure S1 **Specificity of the anti-GABAAR γ2 subunit antibody.**
(PDF)

Figure S2 **Recovery of GABAAR and gephyrin immunofluorescence after 4AP washout.**
(PDF)

Figure S3 **Lateral diffusion of GABAAR with or without FM4–64 labeling.**
(PDF)
