# Correction: Gephyrin-Independent GABA_A_R Mobility and Clustering during Plasticity

**DOI:** 10.1371/annotation/d186036a-da60-47d1-8e3c-7e225a3539ae

**Published:** 2012-05-17

**Authors:** Fumihiro Niwa, Hiroko Bannai, Misa Arizono, Kazumi Fukatsu, Antoine Triller, Katsuhiko Mikoshiba

Corresponding authorship was incorrectly omitted for two authors. The following are corresponding authors on this article: Hiroko Bannai (hbannai@brain.riken.jp), Antoine Triller (triller@biologie.ens.fr), and Katsuhiko Mikoshiba (mikosiba@brain.riken.jp). 

