# Supplementary material for: Gephyrin-Independent GABAAR Mobility and Clustering during Plasticity
Source: PLoS One. 2012 Apr 26;7(4):e36148. doi: 10.1371/journal.pone.0036148 (PMC3338568; doi:10.1371/journal.pone.0036148)
Supplement: Figure S1 — Specificity of the anti-GABAAR γ2 subunit antibody. (PDF) [file pone.0036148.s001.pdf]

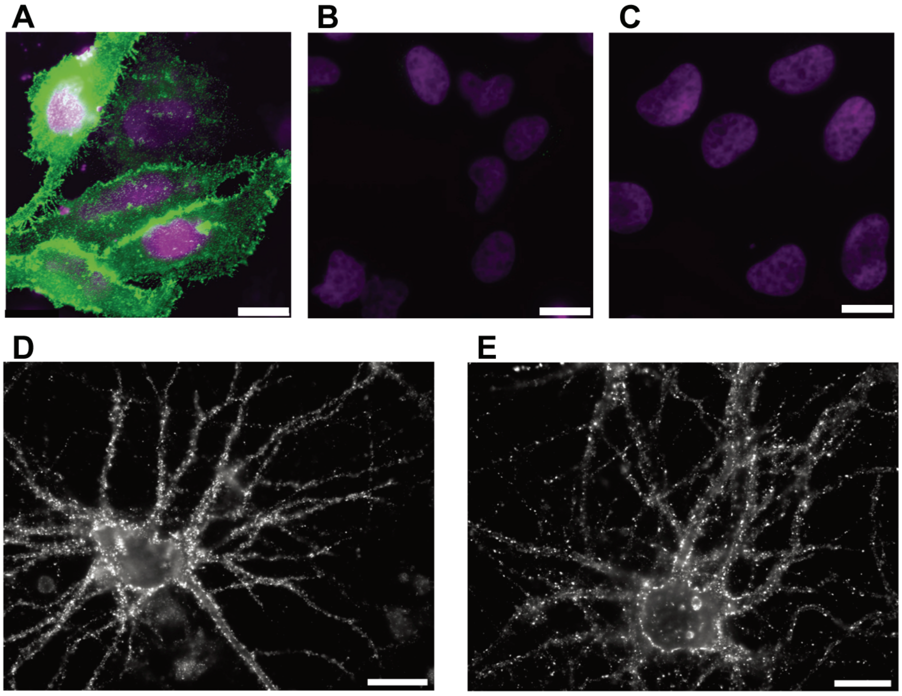

Figure S1. Specificity of the anti-GABA$_A$R γ2 subunit antibody.

A–**C**: Immunocytochemistry with HeLa cells using our custom-made anti-GABA$_A$Rγ2 antibody. Magenta signals indicate nuclei labeled with DAPI. Immunoreactivity (green) was detected in HeLa cells transfected with plasmids encoding GABA$_A$R α1, β3, and γ2 subunits (**A**), but not in non-transfected cells (**B**) and GABA$_A$R-expressing HeLa cells in the absence of the antibody (**C**). **D**, **E**: Immunofluorescence images of endogenous GABA$_A$Rs on cultured hippocampal neurons stained with our custom-made antibody (**D**) and with the antibody used in a previous study [11] (**E**). Scale bars: 20 μm.

Reference

[11] Bannai H, Levi S, Schweizer C, Inoue T, Launey T, et al. (2009) Activity-dependent tuning of inhibitory neurotransmission based on GABA$_A$R diffusion dynamics. Neuron 62: 670-682.
