# Supplementary material for: Gephyrin-Independent GABAAR Mobility and Clustering during Plasticity
Source: PLoS One. 2012 Apr 26;7(4):e36148. doi: 10.1371/journal.pone.0036148 (PMC3338568; doi:10.1371/journal.pone.0036148)
Supplement: Figure S2 — Recovery of GABAAR and gephyrin immunofluorescence after 4AP washout. (PDF) [file pone.0036148.s002.pdf]

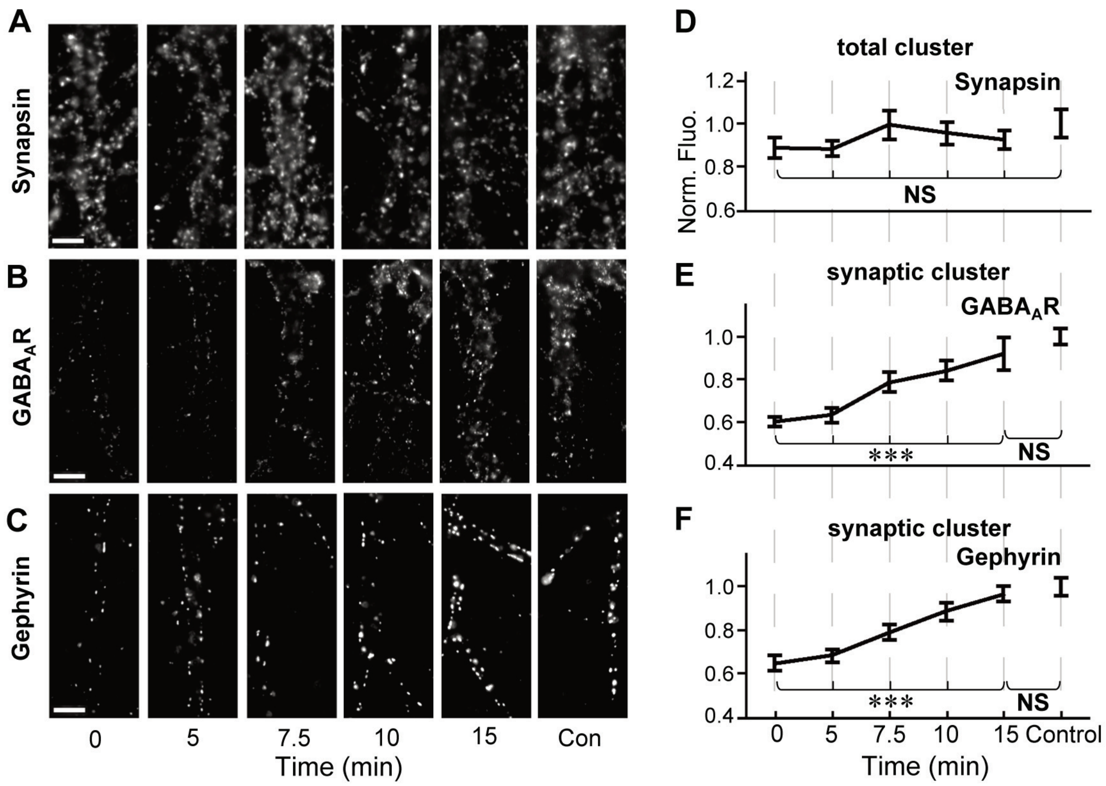

Figure S2. Recovery of $GABA_AR$ and gephyrin immunofluorescence after 4AP washout.

A–**C**: Representative examples of immunofluorescence recovery of synapsin (**A**), $GABA_AR$ (**B**), and gephyrin (**C**) on the dendrites of hippocampal neurons after washout of 4AP. Scale bars: 5 μm.

D–**F**: Time-course plot of recovery of normalized fluorescence intensity (average ± SEM) associated with synapsin (**D**), synaptic $GABA_AR$ (**E**), and synaptic gephyrin (**F**) after washout of 4AP. NS: $p > 0.05$, ***: $p < 0.005$, Tukey's range test in ANOVA, n = 30 cells/condition (3 cultures) for synapsin and $GABA_AR$, n = 40 cells/condition (4 cultures) for gephyrin.
