# Supplementary material for: Gephyrin-Independent GABAAR Mobility and Clustering during Plasticity
Source: PLoS One. 2012 Apr 26;7(4):e36148. doi: 10.1371/journal.pone.0036148 (PMC3338568; doi:10.1371/journal.pone.0036148)
Supplement: Figure S3 — Lateral diffusion of GABAAR with or without FM4–64 labeling. (PDF) [file pone.0036148.s003.pdf]

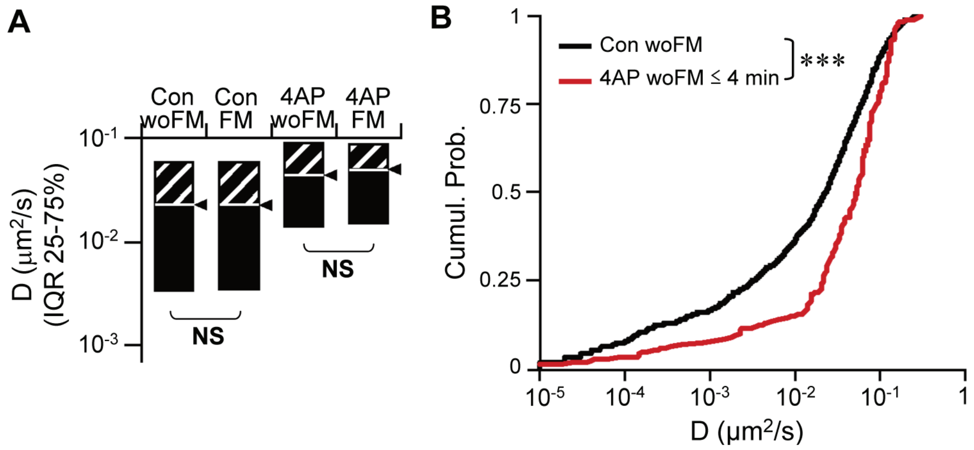

Figure S3. Lateral diffusion of GABA$_A$R with or without FM4-64 labeling.

A: Diffusion coefficients of GABA$_A$R-QDs (median ± IQR) in the absence (Con) and presence (4AP) of 4AP treatment with (FM) or without (woFM) FM4-64 labeling. NS: $p > 0.05$, Mann–Whitney *U* test. The number of GABA$_A$R-QDs analyzed: 1364 QDs for Con woFM; 1183 for Con FM; 1172 for 4AP woFM; 1133 for 4AP FM, from 3 cultures. Note that FM4-64 labeling did not significantly alter the diffusion coefficient. **B**: Cumulative plot of diffusion coefficient for GABA$_A$R-QDs on neurons treated with 4AP for less than or equal to 4 min (4AP woFM ≤ 4 min, red) and control neurons (Con woFM, black) without FM staining. The diffusion of GABA$_A$R-QDs increased within 4 min of 4AP stimulation even in the absence of FM labeling. The number of GABA$_A$R-QDs analyzed: 1364 QDs for Con woFM, 123 for 4AP woFM ≤ 4 min (3 cultures).
